# Case of Large Polypus of the Uterus

**Published:** 1878-07

**Authors:** D. I. McMillan

**Affiliations:** Sunbeam, Ill.


### CASE OF LARGE POLYPUS OF THE UTERUS.

By D. I. McMillan, M. D., Sunbeam, Ill.

I was called, Feb. 20, 1878, to see Mrs. W., who was reported as suffering from labor pains, with something also protruding from the vulva. She was a farmer's wife, 45 years of age, and the mother of six children, the youngest aged 10, some of the oldest married.

I found another physician in attendance, who had, on the day previous, prescribed for what he supposed to be menstrual disorder. At that time she was losing a good deal of blood, and was suffering pain. He supposed that he felt above the pubis an enlarged uterus, which resembled in size and consistence the uterus after labor. Simple remedies were given, and the case dismissed. On this morning he was called in great haste to see her again. She had been suffering most of the night with severe labor pains accompanied by flooding, but as he lived at a distance of five miles he did not see her until two o'clock in the afternoon. He then found a foreign body outside of the vulva, and not satisfied as to its nature nor as to the proper treatment, he requested the counsel of another physician, and I was then sent for. It was eight o'clock when I reached the patient, and found her in the following condition: She had the pallor of death, and seemed completely exsanguine, with an anxious expression of countenance; the pulse was thread-like and very rapid, probably 140 or 150 to to the minute; her extremities were cold and her body bathed in a cold sweat; the tongue had the same bloodless appearance; the respirations were hurried and the eyes protruding, as in suffering from dyspnœa.

On examination, I found an ovoid tumor, nearly as large as a fœtal head, outside the vulva and lying on the bed between the thighs. I say ovoid, but perhaps the best descriptive term would be pear shaped, the upper or stem end of the pear representing the peduncular attachment which passed upward through the vulva. This pedicle was nearly as large as the wrist, or between two and three inches in diameter, and could be traced upward through the os. On account of the large size of this pedicle, which nearly filled the vagina, it could only be traced as far as the finger could reach. The color of the mass was dark red, the pedicle lighter and covered, as it was supposed, with coagulated blood. There was, however, no oozing of blood from any part. In consequence of the external appearance, shape and consistence of the mass, considered in connection with the fact that the womb could not be felt, and that the patient complained of pain when the tumor was handled, we supposed there was partial inversion of the uterus. The darkened appearance we supposed to be caused by venous engorgement from obstruction to the flow of blood through the constricted neck of the womb. The size of the mass was supposed to be owing to the length of time that had elapsed since its extrusion. This took place in the morning, some sixteen hours before, and if there had been any vascular obstruction, the longer this existed the greater would have been the consequent distension and tumefaction.

As we were not satisfied regarding the diagnosis and proper plan of treatment, further advice was requested, and Dr. Frazier, of Viola, was summoned. During the absence of the messenger (a period of several hours, owing to the distance), free stimulation was employed, in order if possible to secure reaction. No measures of this sort had been taken, and the patient was bordering on collapse. To this end alcoholic stimulants were freely used internally and bottles of hot water applied externally. The condition of the patient was not, however, greatly improved by this course. There was the same thready pulse, hurried respiration and coolness of body and extremities.

On Dr. Frazier's arrival he made an examination, and pronounced the mass that we had taken for an inverted uterus, a bloody tumor, with the uterus perhaps partially inverted and enclosed in the blood coagula. He proposed the immediate separation of the uterus and removal of the fungous body. The vitality of the mass was tested by running the finger through the larger portion in different places ; no hæmorrhage followed. He then proceeded to remove the larger portion by twisting it from the pedicle. Considerable force had to be used to detach it. After this was accomplished, the pedicle was attacked. The tissue composing this was very much more dense than that composing the body, and was removed with no little difficulty. Strong traction was made, and the pedicle and fundus of the womb were drawn outside of the vulva. In fact, the tissues composing the pedicle were so nearly like ordinary muscular fiber, that it took considerable discrimination to tell where it ended and uterine fiber commenced. It was detached piecemeal, the finger being used to slit it up and the shreds being peeled off one by one. So firm was the attachment to the fundus that its detachment seemed to require as much force as the removal of muscular tissue from periosteum or bone.

The mass removed, the uterus was easily replaced by a uterine repositor that I happened to have. This could however have been as easily accomplished with a blunt pointed stick. The uterus was perfectly flaccid; no more resistance was experienced than in replacing a prolapsed rectum. No hæmorrhage followed, not even slight oozing of blood. On the site of attachment, where the pedicle was removed, the uterine tissue was blanched, having the same bloodless appearance as the external aspect of the body. This showed how completely the blood had been drained away, since a large vascular tumor had been torn away piecemeal from the uterine surface without hæmorrhage.

The removal of the tumor and replacement of the organ to its normal position gave no relief. The patient was very much exhausted after the operation. A great deal of pain was manifested, especially during the removal of that portion of the pedicle attached to the fundus. The operation over, free stimulation was resumed. Besides external heat and alcoholic stimulants internally, a solution of sulphate of cinchonidia in brandy (quinine not being at hand) was used hypodermically; all, however, without effect. The pulse never grew stronger, but perceptibly weaker, and the patient gradually sank, and died six or eight hours after the operation.

Transfusion of blood was of course greatly needed in her case, but no apparatus of such kind was attainable, not even a syringe. That she died from loss of blood was manifest. Shock, of course, had its effect, but it is my opinion that if a few ounces of blood could have been thrown into her veins immediately after the removal of the tumor, she might be living to-day.

The tumor was, almost certainly, a polypoid growth. Dr. Frazier also, after examining the tumor, and learning the patient's history, concluded that the tumor was a polypus. Her previous history I afterward learned from a lady living in the vicinity. She had been complaining for three or four years of great trouble at the menstrual periods, which were normal as to time, but always excessive and offensive. She was always prostrated and in bed from one to two weeks after each epoch; in fact, so regular was this that she always made preparation for this event by providing for extra changes of linen. Pain was also a prominent symptom during the menstrual flow. She had had an anæmic look for several years. This would indicate that the commencement of the tumor dated back several years, or from the commencement of her uterine trouble.

The composition of the tumor would also indicate its nature. The examination of its structure was made only by the naked eye, and of course could only be superficial. Its entire length, pedicle included was perhaps eight inches. Its weight was probably two, or at most three, pounds. The large portion of the mass was of low vitality. In the language of Dr. Frazier, who examined it the next morning, "It was composed of loose fibrous bands, the interstices filled with clotted blood; over the upper and larger portion was found a membrane more or less tough and resisting." The pedicle was much more dense, as may be readily inferred from the extraordinary difficulty attending its removal. The previous history of the patient, and the character of the growth, both point, therefore, unmistakably to polypus as the real source of the difficulty.

If the character of this tumor had been recognized in the morning, its prompt removal and the replacement of the partially inverted uterus would have been indicated. This, of course, was required fully as much when I saw the patient. I must admit, however, that the resuscitation of the patient seemed most imperative. Although there was no hæmorrhage, and had not been, as far as I could learn, after the expulsion of the polypus, it was nevertheless quite plain that the woman was dying from loss of blood and shock. The dragging down of the womb by the tumor had of course to be relieved, as this was increasing the prostration of the patient, although hæmorrhage had ceased hours before.

It is a question in my mind whether the womb should have been pulled down and the pedicle torn from its uterine attachment, as this produced pain and increased the prostration. Had the patient lived she might also have suffered from hæmorrhage at the seat of the implantation of the pedicle. The better plan would have been (it seems to me), to have ligated the pedicle, cut the tumor off, and then returned the womb to its normal position. As has been stated, it was not easy to discriminate between pedicle and uterus, so much did they resemble each other in consistency and appearance, great care being required to avoid tearing away a portion of the womb.

